# Evaluation of Sodium Alginate Stabilized Nanoparticles and Antibiotics against Drug Resistant *Escherichia coli* Isolated from Gut of Houbara Bustard Bird

**DOI:** 10.1155/2022/7627759

**Published:** 2022-09-12

**Authors:** Afshan Muneer, Santosh Kumar, Amjad Islam Aqib, Shanza Rauf Khan, Syed Qaswar Ali Shah, Iqra Zaheer, Tauseef ur Rehman, Asghar Abbas, Kashif Hussain, Atif Rehman, Muhammad Nadeem, Maheen Murtaza, Ahmad Waseem

**Affiliations:** ^1^Department of Zoology, Cholistan University of Veterinary and Animal Sciences, Bahawalpur 63100, Pakistan; ^2^Department of Medicine, Cholistan University of Veterinary and Animal Sciences, Bahawalpur 63100, Pakistan; ^3^Department of Chemistry, University of Agriculture Faisalabad, 38000, Pakistan; ^4^Department of Pathology, University of Agriculture Faisalabad, 38000, Pakistan; ^5^Department of Parasitology, The Islamia University of Bahawalpur, Pakistan; ^6^Faculty of Veterinary and Animal Sciences, Muhammad Nawaz Sharif University of Agriculture, Multan, Pakistan; ^7^Department of Pathology, PMAS-Arid Agriculture University Rawalpindi Sub Campus Khushab, Pakistan; ^8^Houbara Foundation International, Lal Sohanra Park, Bahawalpur 63100, Pakistan

## Abstract

Alternative approaches and/or modified approaches to tackle resistance in gut microbes are need of the hour. The current study was planned to find the resistance modulation and toxicity potential of sodium alginate stabilized MgO nanoparticles and antibiotics against *Escherichia coli* (*E. coli*) isolated from the gut of Houbara bustard bird (*n* = 105 fecal samples). The preparations consisted of gel stabilized ampicillin (G+A), gel stabilized MgO and ampicillin (G+M+A), gel stabilized MgO and cefoxitin (G+M+C), gel stabilized tylosin (G+T), gel stabilized MgO and tylosin (G+M+T), and gel stabilized MgO (M+G). The fecal samples showed 53% (56/105) prevalence of *E. coli* which was found to be significantly (*p* < 0.05) associated with most of the assumed factors and resistant to multiple drugs. G+M+T showed the lowest (4.883 ± 0.00*μ*g/mL) minimum inhibitory concentration (MIC) followed G+M+C, G+M+A, G+A, M+G, and G+T. Significant reduction (*p* < 0.05) in MIC with respect to incubation interval found at the 16^th^ hr for G+M+A, G+A, and G+M+C that further remained nonsignificant (*p* > 0.05) onwards until the 24^th^ hr of incubation. In the case of G+T and M+G, significant reduction in MIC was found at the 20^th^ hr and 24^th^ hr of incubation. Ecotoxicology and histopathology trials on snails showed mild changes in MICs of the preparations. The study thus concluded increasing drug resistance in *E*. *coli* of houbara bird while sodium alginate stabilized MgO nanoparticles and antibiotics were effective alternative antibacterial composites with mild toxicity.

## 1. Introduction

*Escherichia coli* is the main bacteria carried by migratory birds which exist as infectious microorganisms in the intestine of avian species [1]. These bacteria are equally becoming major pathogens same as Salmonella from poultry [2]. Infectious *E. coli* can cause wider range of diseases in calves [3] and deterioration of products like meat [4] in addition to intestinal disorders. Migratory birds on the other hand have a big impact on the spread of antibiotic-resistant bacteria and ARGs (antibiotic resistance genes) [5]. Among several other factors that contribute to the antibiotic resistance are the continued and improper antimicrobial use of the drugs [6] giving rise to multiple drug resistance in *E.coli* which has also been reported in migratory birds worldwide [7]. The Houbara bustard (*Chlamydotis undulate*) or Tiloor (local name in Pakistan) is one of these migratory birds which are considered threatened species red listed by IUCN (International Union for Conservation of Nature) at risk [8]. Population of Houbara bustard ranged between 78960 and 97000 in the year 2014 whereas there are reports in its declining population at the rate of 30-49% [9].

Alternative approaches to tackle pathogens include, however are not limited to conventional plants [10], nanoparticles, peptides, prebiotics, and probiotics. Control of gut microbes and improved health are achievable milestones for better health and production of birds [11] and nanoparticles. Currently, nanotechnology plays an important role in advances in medicine and pharmaceuticals [12]. In addition to higher reactivity, greater surface-to-volume ratios, stability, bioactivity, bioavailability, controlled particle sizes, and controlled release of drugs make these particles unique in physicochemical properties [13]. The combination of nanoparticles with antibiotics, antimicrobial peptides, and essential oils has shown to reduce possible toxic effects of nanoparticles on mammalian cells in recent studies [14, 15].

It is however important to find modified approaches to enhance antibacterial activity with minimized toxicity. Stabilization of preparations is one of the appreciable approaches for an extended period. Sodium alginate gel has many uses in biomedical sciences as an excipient of drug delivery systems, tissue engineering, preservative in food products, and nanomedicines in various forms [16]. The gel itself has shown antibacterial potential in various previous studies while its uses also have been found beneficial in various wound dressings to avoid secondary bacterial infection [17]. In nanomedicines, the gel has been used as dendrimers, emulsions, lipids, nanocrystals, nanoparticles, polymeric nanoparticles, and micelles [18]. The biodegradability and biocompatibility along with very mild toxicity make its application in broader phases of various industries. Thus, there is a dire need to enhance the activity of antibiotics and non-antibiotic alternatives with reduced dosage and negligible toxicity [19]. This study focused on the evaluation of antibacterial activity of MgO nanoparticles and antibiotics stabilized in sodium alginate gel along with investigation of toxicity parameters.

## 2. Materials and Methodology

### 2.1. Preparation of Sodium Alginate Stabilized MgO Nanoparticles and Antibiotics

A hydrothermal method was used to synthesize MgO nanoparticles in the presence of a surfactant. MgCL_2_.6H_2_O (4 grams) was dissolved in 40 mL of distilled water which was further kept at constant magnetic stirring for 4 hrs at room temperature. Sodium dodecyl sulphate (4 g) was further added in MgCl_2_ solution. It was required to maintain 12 pH which was executed by adding 20 mL of 2.5 M NaOH drop by drop to the reaction mixture [20]. After stirring (4 hrs), the white suspension was obtained which was further heated for 6 hrs at 140°C. The white precipitate was washed and centrifuged and subsequently dried for 24 hours at 60°C in a thermoelectric oven. The ground precipitates obtained were further calcined at 450°C/3 hrs [21]. A 2% (m/v) Na-alginate solution and 2% (m/v) gelatin solutions were prepared in water. Both solutions were mixed in 80 : 20 ratio (sodium alginate: gelatin) at 500 rpm for 2 hrs by using a mechanical stirrer to yield sodium alginate gel (abbreviated in the manuscript as G). To stabilize MgO within gel, 1.5 g of MgO nanoparticles were incorporated in 20 mL of gel and stirred for 4 hrs at 500 rpm. The drug (0.035 grams) was dissolved in distilled water to finally prepare a 20 mL solution. A mixture of 20 mL of gel (sodium alginate) and 20 mL of ampicillin solution was mixed and stirred at 500 rpm for 4 hrs. The mixture was stirred at 500 rpm for 4 hrs which was then dried and ground.

Energy Dispersive X-ray Spectroscopy (EDX) of nanoparticles was carried out. A 30 kV Quanta 250 was operated to find SEM (Scanning electron microscopy) images for MgO nanoparticles. The composites were formulated in powder form and were checked through instruments as final judgment. The products finally prepared were M + G = MgO stabilized in gel, G + M + T = MgO and tylosin stabilized in gel, G + M + C = MgO and cefoxitin both stabilized in gel, G + M + A = MgO and ampicillin stabilized in gel, G + T = tylosin stabilized in gel, G + A = ampicillin stabilized in the gel.

### 2.2. Toxicity of Sodium Alginate Stabilized Nanoparticles and Antibiotics

For toxicity evaluation, *n* = 65 healthy and active land snails were taken from a garden where chemicals and pesticides were not used previously. There were 07 groups of snails including the control negative group. Each treatment group was further divided into two groups: (1) Group receiving dose at 1 × MIC and (2) Group receiving 10 × MIC. Each group was having *n* = 05 snails allocated on random basis (Table 1). A 50 *μ*L of the solution was poured on the anterior side of the snail's mouth portion. Snails were kept off feed for 05 days and dead were immediately processed for histopathology [20].

### 2.3. Histopathology

The snails were dissected while digestive glands were collected in Bouin's fluid until further used. After deparaffinizing of the fixed sections, the 5 mm thick sections were hydrated and stained in hematoxylin for 15 minutes. It was counter stained with a 1% Eosin solution for two minutes after washing with water. Dehydration was accomplished with alcohol, which was then cleaned in xylene before being mounted on clean microscope glass with Canada balsam and lastly covered with thin cover slides Cell disintegration, unusual nuclei ranging from karyolysis to severe karyorrhexis, full pyknosis, abundant vacuolation, and frequent dark granulation were considered main pathological alterations [22].

### 2.4. Gut (Fecal) Sample Collection

To carry out sampling from the bird, prior consent was taken from the Houbara International Foundation Lal Suhanra Park, Bahwalpur, Pakistan while ethical consideration was observed as per standard protocols. A total of *n* = 105 fecal samples from cloaca were aseptically collected (*n* = 70 male, *n* = 35 female) at different time intervals [23]. A sterile cotton swab moistened with normal saline was inserted into the cloacae of the Houbara bustard bird. Once the cloaca swabs were collected, the birds were released [24]. The samples were immediately shifted to the laboratory of Houbara Foundation International, Lal Suhanra Park, Bahawalpur, Pakistan which was situated at a few yards from the sampling area. The samples were preserved in an ice box maintaining 4°C which were finally shifted to the Central Diagnostic Laboratory (CDL), Cholistan University of Veterinary and Animal Sciences, Bahawalpur. A questionnaire was also filled in with necessary information about bird and its housing to analyze the association of assumed risk factors with prevalence of *E. coli*.

### 2.5. Isolation of *E. Coli*

Fecal samples were incubated in sterile nutrient broth overnight at 37°C. The incubated samples were homogenized on a stirrer, and sterile swabs were dipped in homogenized material. These swabs were gently spread on blood agar and incubated at 37°C/24 hrs. The colonies obtained were further streaked on MacConkey agar and incubated for 24 hrs at 37°C. A series of biochemical tests were performed to identify *E.coli* [25] following standard protocols described in Bergey's Manual of determinative bacteriology.

### 2.6. Molecular Identification of *E. Coli*

**Figure 1:**
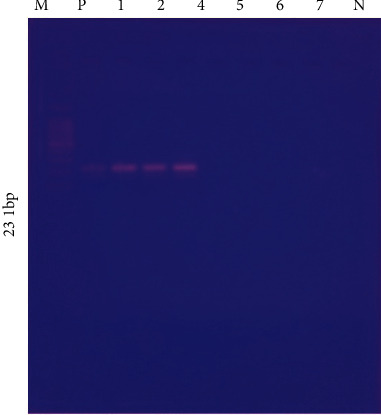
Agarose gel picture of amplicons of polymerase chain reaction used for identification of *E. coli* isolated from cloaca of the Houbara bustard. M: marker 1000 bp, wells 1–7 sample at 231 bp, +ve: positive control, −ve: negative control.

The isolates identified by biochemical methods were also subjected to molecular confirmation using PCR. The specific primers for 23S rRNA gene of 231 bp (E23S-F: ATCAACCGATTCCCCAGT; E23S-R: TCACTATCGGTCAGTCAGGAG) were used in PCR to target *E. coli* from biochemically identified bacterial cultures. During the temperature cycling, a short period of 95°C was set for 3 minutes, followed by a longer period of 95°C for 1 minute for 335 cycles, followed by a final extension stage of 72°C for 7 minutes. The amplicons were run on 2% agarose gel kept at 100 volts for 1 hr through electrophoresis equipment, and finally the gel was visualized in UV light [26] (Figure 1).

### 2.7. Antibiotic Susceptibility of *E. Coli*

A total of *n* = 10 antibiotics (enrofloxacin, fusidic acid, ciprofloxacin, sulfamethoxazole-trimethoprim, levofloxacin, chloramphenicol, vancomycin, gentamicin, linezolid, and cefoxitin) were tested against *E. coli* to find the status of its susceptibility. Kirby Bauer disc diffusion method was applied following guidelines of the Clinical and Laboratory Standard Institute (CLSI). Briefly, fresh growth of *E. coli* adjusted at 1 − 1.5 × 10^8^ CFU/mL was spread over sterile Mueller Hinton agar. The antibiotic discs were aseptically and gently punched at equal distances from each other. Following incubation at 37°C for 20-24 hrs, zones of inhibitions were measured and compared with standards provided in CLSI, [27].

### 2.8. *In Vitro* Antibacterial Activity of Sodium Alginate Stabilized Products

To assess the potential of sodium alginate stabilized products, well diffusion method was applied as an empirical method for estimation of antibacterial activity. The broth microdilution method was applied to find a minimum inhibitory concentration (MIC) of different preparations.

#### 2.8.1. Agar Well Diffusion Method

Sterile Mueller Hinton agar was dug up 6-8 mm wide using a well borer. A fresh culture of *E. coli* adjusted at 1 − 1.5 × 10^8^ CFU/mL was spread over Mueller Hinton agar. A 50 *μ*L of 0.01 gm/mL of sodium alginate stabilized products were poured. Following 24 hrs incubation at 37°C, zones of inhibitions (ZOIs) produced by the products against *E. coli* were measured by vernier calipers [28].

#### 2.8.2. Minimum Inhibitory Concentration (MIC)

Two-dimensional outcomes were intended on this section: (1) comparison of MIC among different preparations and (2) comparison of incubation periods within each preparation. For this purpose, 96 well titration plates were filled in with Mueller Hinton broth. The preparations were serially diluted by the two-fold dilution method. Finally, the fresh growth of *E. coli* set at 10^5^ CFU/mL was poured into each well except for negative control. The well designated as negative control consisted of only broth while that of positive control contained broth and culture. Optical density values were taken at 695 nm wavelength through an ELISA reader at 0 hr, 4 hrs, 8 hrs, 12 hrs, 16 hrs, 20 hrs, and 24 hrs. The values taken were compared with 0 hr incubation to find the net OD value. Net OD value was considered to find inhibition of growth at various concentrations. The minimum concentration of preparation that inhibited the growth of bacteria was declared as MIC [27, 28].

### 2.9. Statistical Analysis

Univariate analysis was carried out for data on prevalence while chi-Square analysis was done to find the association of assumed risk factors with the prevalence of *E. coli.* Parametric tests like analysis of variance (ANOVA) coupled with Tukey's test were applied at 5% probability to compare means of the zones of inhibition and MIC values of sodium alginate stabilized products. SPSS version 22 of the statistical computer program was applied to analyze data. Prevalence was calculated as per the formula [23]:

(1) $$\begin{array}{c} \text{Prevalence percentage }\left(\%\right)=\frac{\text{No of sample positive for }E.coli}{\text{Total No of samples}}\times 100. \end{array}$$

## 3. Results

### 3.1. Characterization of Nanoparticles

**Figure 2:**
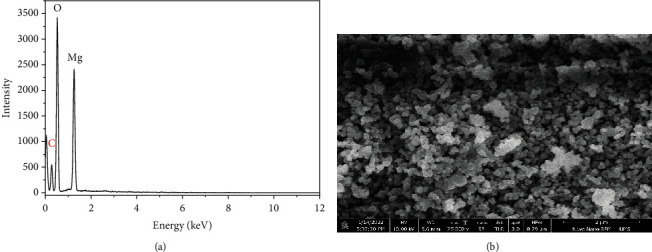
Characterization of MgO nanoparticles: (a) SEM images of MgO nanoparticles synthesized by the hydrothermal method and (b) XRD pattern of synthesis of MgO nanoparticles.

Three peaks situated at 1.26, 0.52, and 0.28 keV were identified in pattern. Two peaks located at 1.26 and 0.52 keV matched with Mg and O, respectively. One extra peak of carbon was identified at 0.28 keV. This peak was due to carbon based sample stacking tape. Both of the peaks (1.26 and 0.52 keV) were due to K*α* transitions in Mg and O, respectively. K*α* transition was happened when electrons were dropped from L (*n* = 2) to K (*n* = 1) shell. The analysis of EDX data showed that Mg and O were successfully incorporated, and MgO was synthesized (Figure 2(a)). Overall morphology of product is monodisperse because nearly spherical shaped nanoparticles were observed from this figure. The boundaries of nanoparticles were defined. Nanoparticles were not agglomerated but found in dispersed form. The size of nanoparticles was ranged from 80 to 150 nm.

### 3.2. Ecotoxicity Analysis

The sodium alginate stabilized antibiotics and MgO nanoparticles showed direct proportion of mortality with increasing concentration of products (Table 1). There was a 20% difference in mortality of all products except those having nanoparticles and antibiotics simultaneously stabilized in sodium alginate (G+M+A, G+M+C, G+M+T). Highest mortality was noted in gel stabilized antibiotics (G+A, G+T) and gel stabilized nanoparticle (M+G) at both of 1 × MIC and 10 × MIC concentrations for these products.

### 3.3. Histopathology

**Figure 3:**
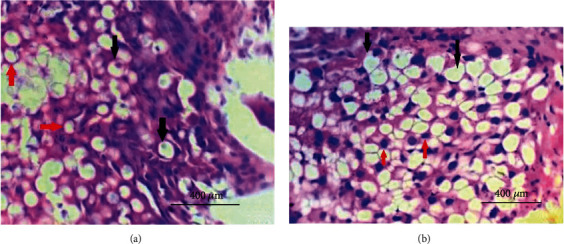
Microscopic lesions produced by sodium alginate stabilized composites in digestive glands of snail (stained by H&E staining method). (a) Treatment level 4.883-26.04 *μ*g/mL of sodium alginate stabilized nanoparticles and antibiotics (at 400 X): shows vacuolar degeneration of digestive gland cells marked by the marginal nucleus (red arrows) while there are some normal cells presented with central and lighter and bigger nucleus (black arrows). (b) 10 Times the MIC of products (48.83-260.4 *μ*g/mL). Sodium alginate stabilized composites treated digestive gland (at400 X): shows vacuolar degeneration (black arrows) and pyknotic nuclei indicating cellular degeneration (red arrows).

Snails treated with 1×MIC of different concentrations of sodium alginate stabilized products were observed to have induced microscopic alterations at several locations of the digestive tract (Figure 3(a)). The digestive glands located in the region appeared degenerated marked by the development of excretory cells. Vacuolation, and the release of secretory granules. The basophilic infiltration was evident through the parenchyma of the digestive glands. Surrounding connective tissue also seemed disintegrated, and the lumen appeared irregular and fluid filled. Pyknotic nuclei were observed in the parenchyma and interstitial tissue (Figure 3(b)).

### 3.4. Prevalence of *E. Coli* and Its Associated Risk Factors

The current study found 53% (56/105) prevalence of *E. coli* from fecal samples of the Houbara bustard bird. Female birds presented 72.85% while male birds showed a 57.14% prevalence of *E. coli* (Table 2). Association of risk factors with prevalence of *E. coli* was found significant (*p* < 0.05) with feeding system, season, and gastrointestinal parasites. On the other hand, age, gender, housing system, use of antibiotics, and types of antibiotics showed nonsignificant (*p* > 0.05) associations.

### 3.5. Antibiogram

The study found increasing trends of antibiotic resistance in *E. coli* (Table 3). The antibiogram showed 40% of *E. coli* resistant to fusidic acid and cefoxitin while 30% showed resistance against ciprofloxacin and vancomycin. Levofloxacin, chloramphenicol, linezolid, and sulfamethoxazole-trimethoprim faced 20-25% resistance while against gentamicin there were only 10% resistant isolates. None of the isolates were found resistant to enrofloxacin in this study. On the other hand, considerable intermediate susceptible isolates were noted against different antibiotics in that there were 50% of isolates intermediate susceptible against levofloxacin, while 30% showed the same response against vancomycin, gentamicin, and linezolid. The study also noted 20-25% of isolates falling in intermediate susceptible category against enrofloxacin, ciprofloxacin, sulfamethoxazole-trimethoprim, chloramphenicol, and cefoxitin.

### 3.6. Comparison of Different Preparations for Minimum Inhibitory Concentration (*μ*g/mL)

The study showed a significant difference (*p* < 0.05) in MIC values among different treatments following 24 hrs incubation (Table 4). G+M+T showed lowest MIC (4.883 ± 0.000*μ*g/mL) followed by G+M+C (6.51 ± 2.82*μ*g/mL), G+M+A (13.02 ± 5.64*μ*g/mL), G+A (13.02 ± 5.64*μ*g/mL), M+G (16.28 ± 5.64*μ*g/mL) and G+T (26.04 ± 11.28*μ*g/mL). G+M+A showed a non-significant difference (*p* < 0.05) in MIC compared to those of G+A, G+T, and G+M+A while a significant difference (*p* < 0.05) was noted in comparison with the MICs of G+M+T and M+G. However, all other preparations did not show a significant difference (*p* < 0.05) among each other. There was a higher standard deviation in some preparations, a possible reason for a nonsignificant difference. It was also noted from this study that comparison of MICs among different preparations remained variable at different time intervals of incubation.

**Figure 4:**
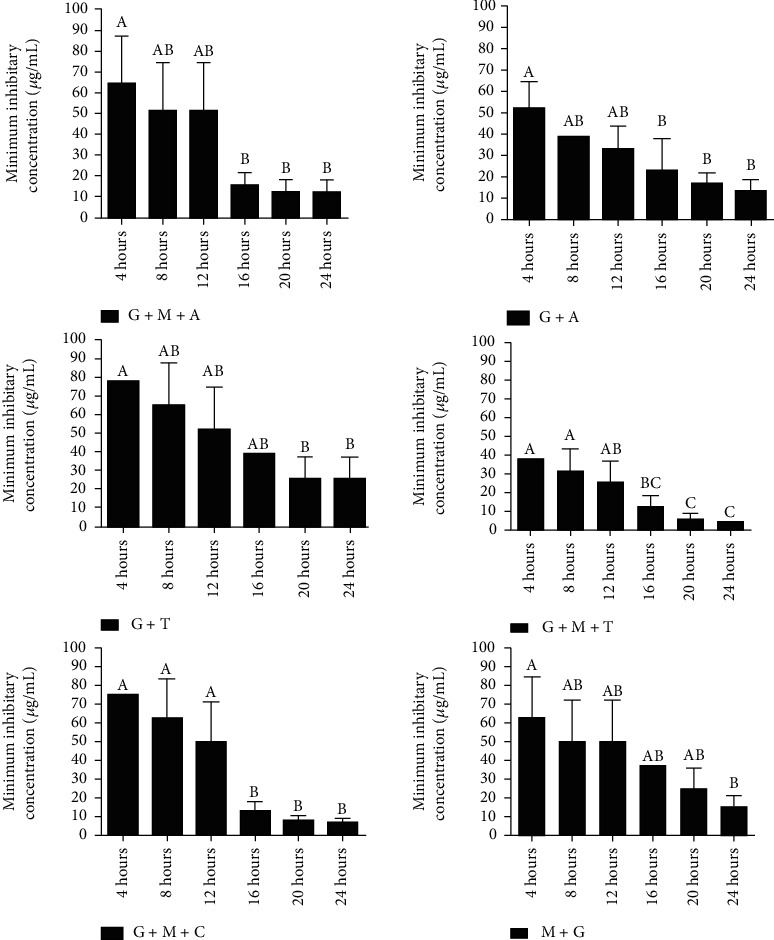
Minimum inhibitory concentration of different treatments/s at different time intervals G + M + A = MgO and ampicillin stabilized in sodium alginate gel; G + A = ampicillin stabilized in sodium alginate gel; G + T = Tylosine stabilized in sodium alginate gel; G + M + T = MgO and tylosin stabilized in sodium alginate gel; and M + G = MgO stabilized in sodium alginate gel.

The maximum antibacterial activity at earlier incubation with minimum concentrations was found promising in this study. The current study showed that the response of G+M+A, G+A, M+G, and G+T against *E. coli* remained similar for maximum antibacterial activity at the earliest among incubation periods (Figure 4). A significant reduction (*p* < 0.05) in MIC was noted at the 8^th^ hr of incubation when compared with that of 4^th^ hr incubation. On the other hand, G+M+T and G+M+C showed a significant reduction in MIC at the 12^th^ hr incubation. Moreover, further significant reduction in MIC was noted only in the case of G+M+T at 16^th^ hr of incubation. This fact indicated that depending upon the availability of time, maximum antibacterial activity at the lowest concentration could be achieved.

## 4. Discussion

### 4.1. Characterization of Nanoparticles

The preparation method of MgO nanoparticles using the hydrogel method in the current was in line with the findings of Duong et al. [29]. Moreover, the findings of SEM analysis in the current study were in line with those of Radhi and Jasim [30]. The findings of XRD and SEM of current study were also in line with those of Zaheer et al. [20] who reported 30-80 nm size, without any aggression and proved to be compact. In addition, miller indices in XRD findings of Zaheer et al. [20] were like those in current study while the sharp peaks indicated synthesized product as crystalline.

### 4.2. Ecotoxicity Evaluation

The toxicity analysis of our study was in line with the findings of Zaheer et al. [20] who conducted a trial using similarly snail as the experimental animal. Caixeta et al. [31] also reported results similar to the findings of the current study. A limited number of studies evaluate the hazards of iron oxide nanoparticle treatment to terrestrial invertebrates [32, 33]. Additionally, MgO nanoparticles are listed among FDA-approved materials for safer use. The mechanism of toxicity in insects is yet to be fully understood while the known attribute so far is the production of reactive oxygen species that lead to DNA damage and eventually death of the cell due to higher alkalinity [34]. Some studies also report toxicity due to the slowdown of cellular functions of host cells leading to the death of cells [35].

The fact that snails have a propensity to bioaccumulate nanomaterials, as well as the fact that they are an important element of land and aquatic ecosystems, makes them an excellent model for assessing the ecotoxicity of nanomaterials [20]. Otludil and Ayaz [36] confered increase in the concentration of CuSO4 and the exposure times as directly proportion to the potential of the lesions and their severity in the tissue. The physical properties and chemical composition of nanomaterials determine their molluscicide activity against snails as well as their environmental transformation [31]. Based on biochemical analyses of animal sera, ZnO nanoparticles were shown to be safe during *in vivo* testing [37]. The same results were obtained in humans post 5 days exposure of ZnO nanoparticles to the dermal layers [38].

### 4.3. Prevalence and Associated Risk Factors

The prevalence of *E.coli* found in the current study was in line with those of Literak et al. [39] who reported 57.9% prevalence but contrary to the current study lower prevalence was noted by Zurfluh et al. [40] and higher was reported by Shobrak and Abo-Amer [41]. Similarly, higher prevalence, i.e., 85.7% in Portugal was reported by Rashid et al. [42]. Meanwhile, the prevalence of *E.coli* lower than that of current study was reported by Dotto et al. [43] and Foti et al. [44] who reported 24.31% of positive cases in Italy. The difference in the prevalence of various regions may be described based on the risk factors at various sampling areas like hygienic conditions and prevention measures. Contrary to the findings of our study, Nguyen et al. [45] reported significant (*p* < 0.05) association of use antibiotics with *E.coli*. However, Sarba et al. [46] showed age, health status, and diarrhea to be significantly associated (*p* < 0.05) risk factors. Majhi et al. [47] concluded season as a significant influencing factor for health of the birds. They reported 57% prevalence during the rainy season, 39% during summer, and 19% during the winter season. Rahman et al. [48] recorded colibacillosis cases across all seasons of the year with the highest rate occurring during the summer season at the BRAC Poultry Disease Diagnostic Centre, Gazipur, Bangladesh. Hermans and Morgan [49] reported high disease prevalence in the United Kingdom from October to February. Contradictions in results of current study with those of the previous studies might be due to different locations and seasons.

### 4.4. Antibiogram of *E. Coli* and Response of Different Preparations

Findings of the current study were found in line with those of Anwar et al. [50] and Yu et al. [51]. Infectious *E.coli* of human and animal origin showing resistance to enrofloxacin, ampicillin, gentamicin, penicillin, and ciprofloxacin has also been reported by Hemmatinezhad et al. [52]; Ranjbar et al. [53]. Response against chloramphenicol was found to be 19.2% [54] while there were 100, 91.67, 100, 100, 66.67, and 66.67% of *E. coli* resistant against vancomycin, cefotaxime, tetracycline, ciprofloxacin, amoxicillin, and gentamicin, respectively, [4].

Nano-antibiotics were supposed to possess antimicrobial properties on their own or may enhance the efficacy of conventional antibiotics, both of which have the capability of controlling bacterial infections both *in vitro* and *in vivo* [55]. Different studies have shown that metal nanoparticles combined with antibiotics have enhanced antibacterial activity and anti-MRSA (methicillin-resistant *S. aureus*) activities than nonpolymerized penicillin or N-methylthio *β*-lactams [56]. Similarly, Allahverdiyev et al. [57] reported a combination of amoxicillin with silver nanoparticles as an effective resistance modulator for drug-resistant *E.coli.* Similar findings were reported by Li et al. [15] who noticed high potency against *E.coli.* In another study, superparamagnetic iron oxide nanoparticles (SIONPs) at 5 mg/mL presented potential anti-biofilm expressions on biofilms produced by both Gram-negative and Gram-positive bacteria [58].

## 5. Conclusion

This study concluded that multiple drug resistant *E. coli* from gut of the Houbara bustard was a prevalent pathogen along with a significant association of most of the assumed risk factors. Pathogens demonstrated an increasing trend of resistance to the antibiotics. However, the resistance modulation was found prominent upon the use of sodium alginate stabilized MgO nanoparticles and antibiotics. The preparations showed significant antibacterial activity at the earliest hours of incubation that can be considered in case of outbreaks. Histopathology parameters concluded mild toxicities thus presenting safer use of the composites. This study thus proposes further *in-vivo* trials on composites and documentation of their efficacy, safety, and stability parameters.

## Figures and Tables

**Table 1:** Mortality percentages in snails at various concentrations of preparations.

Product name	Concentration used		Mortality at 1 × MIC (M1)	Mortality at 10 × MIC (M2)	% Difference in mortality % (M2-M1)
	1 × MIC (M1) (*μ*g/mL)	10 × MIC (M2) (*μ*g/mL)			
G+M+A	13.02	130.2	(2/5) 40%	(2/5) 40%	0
G+A	13.02	130.2	(2/5) 40%	(3/5) 60%	20
G+T	26.04	260.4	(3/5) 60%	(4/5) 80%	20
G+M+C	6.51	65.1	(2/5) 40%	(2/5) 40%	0
G+M+T	4.883	48.83	(1/5) 20%	(1/5) 20%	0
M+G	16.28	162.8	(2/5) 40%	(3/5) 60%	20
Control negative	Placebo		(1/5) 20%		

MIC: Minimum inhibitory concentration; values in brackets shows ratio of died from total tested, M1: mortality at 1 × MIC concentration, M2: mortality at 10 × MIC, M + G = MgO stabilized in gel, G + M + T = MgO and tylosin both stabilized in gel, G + M + C = MgO and cefoxitin both stabilized in gel, G + M + A = MgO and ampicillin both stabilized in gel, G + T = tylosin stabilized in gel, G + A = ampicillin stabilized in gel.

**Table 2:** Risk factors analysis of *E. coli* isolated from the gut of the Houbara bustard.

Variable	Levels	Screened	Positive	Prevalence (%)	*P* value	CI 95%	
						Lower	Upper
Gender	Male	35	20	57.14	0.105	40.85	72.01
	Female	70	51	72.85		61.46	81.88

Age	0-6 M	15	10	66.66	0.253	41.72	84.83
	7-12 M	36	28	77.77		61.92	88.29
	Above 1 Y	54	33	61.11		47.79	72.96

Housing system	Natural environment provision	67	41	61.19	0.062	49.22	71.95
	Pen	38	30	78.94		63.66	88.93

Feeding system	Poultry feed	38	16	42.10	<0.01	27.86	57.81
	Poultry feed plus scavenger	67	55	82.08		63.66	89.45

Season	Spring	40	25	62.5	0.016	47.03	75.78
	Winter	30	28	93.33		78.67	98.15
	Summer	35	18	51.42		35.57	67.01

GI parasites	Yes	44	35	79.54	<0.01	65.50	88.85
	No	61	26	42.62		31.01	55.10

Use of antibiotics	Frequent	25	17	68	0.688	48.41	82.79
	Occasional	30	22	73.33		55.55	85.81
	No use	50	32	64		50.14	75.86

CI: Confidence interval, *p* < 0.05 indicate significant association.

**Table 3:** Antibiotic susceptibility pattern of *E. coli* against different antibiotics.

Antibiotic name (abbreviation and potency)	Resistant (%)	Intermediate (%)	Sensitive (%)
Enrofloxacin (ENR 5 *μ*g)	0	25	75
Fusidic acid (FA 10 *μ*g)	40	10	50
Ciprofloxacin (CIP 5 *μ*g)	30	20	60
Septran (S^∗^T 25 *μ*g)	20	25	25
Levofloxacin (LEV 5 *μ*g)	25	50	25
Chloramphenicol (C 30 *μ*g)	25	25	50
Vancomycin (VAN 30 *μ*g)	30	30	30
Gentamicin (CN 10 *μ*g)	10	30	60
Linezolid (LNZ 30 *μ*g)	20	30	50
Cefoxitin (C^∗^T 30 *μ*g)	40	20	40

**Table 4:** Minimum Inhibitory concentrations (*μ*g/mL) of different preparations against *E. coli.*

Preparations	Time intervals of incubation					
	4 hrs (mean ± SD)	8 hrs (mean ± SD)	12 hrs (mean ± SD)	16 hrs (mean ± SD)	20 hrs (mean ± SD)	24 hrs (mean ± SD)
G+M+A	65.1 ± 22.6^a^	52.1 ± 22.6^a^	52.1 ± 22.6^a^	16.28 ± 5.64^a^	13.02 ± 5.64^a^	13.02 ± 5.64^a^
G+A	52.1 ± 22.6^a^	39.06 ± 0.00^a^	32.55 ± 11.28^a^	22.79 ± 14.92^a^	16.28 ± 5.64^a^	13.02 ± 5.64^ab^
G+T	78.13 ± 0.00^a^	65.1 ± 2.6^a^	52.1 ± 22.6^a^	39.06 ± 0.00^ab^	26.04 ± 11.28^a^	26.04 ± 11.28^ab^
G+M+C	78.13 ± 0.00^a^	65.1 ± 22.6^a^	52.1 ± 22.6^a^	13.02 ± 5.64^b^	8.14 ± 2.82^a^	6.51 ± 2.82^ab^
G+M+T	39.06 ± 0.00^a^	32.55 ± 11.28^a^	26.04 ± 11.28^a^	13.02 ± 5.64^b^	6.51 ± 2.82^a^	4.883 ± 0.000^b^
M+G	65.1 ± 22.6^a^	52.1 ± 22.6^a^	52.1 ± 22.6^a^	39.06 ± 0.00^b^	26.04 ± 11.28^a^	16.28 ± 5.64^b^

Different superscripts within a column indicate a significant difference (*p* < 0.05), SD = standard deviation; M + G = MgO stabilized in gel, G + M + T = MgO and tylosin simultaneously stabilized in gel, G + M + C = MgO and cefoxitin both stabilized in gel, G + M + A = MgO and ampicillin simultaneously stabilized in gel, G + T = tylosin stabilized in gel, G + A = ampicillin stabilized in gel.

## Data Availability

No data is used.
